# Case of a Fungous Enlargement of the Extremity of the Female Urethra; with Remarks

**Published:** 1792

**Authors:** T. Hughes

**Affiliations:** Surgeon at Stroud Water in Gloucestershire.


### IV. *Case of a fungous Enlargement of the Extremity of the female Urethra; with Remarks. By Mr.* T. Hughes, *Surgeon at Stroud Water in Gloucestershire.*

IN the month of May, 1769, my advice was desired for a young lady in the eleventh year of her age, and of a very thin habit of body, for a disorder first taken notice of above three years before, within a few days after her having taken a purge which had operated smartly. A surgeon, who was consulted soon after the disease appeared, had given it as his opinion that it was a prolapsus uteri, and accordingly advised a bit of cork to be worn as a pessary. This occasioned so much pain, that it was laid aside after having been used a few days; from which time she wore only a bolster of linen sustained by a bandage. From the beginning there had been a discharge from the part, which her friends imagined kept her thin: they shewed me her linen, and I observed that it was stained to a considerable extent with a bloody serum. She sometimes complained of pain in the part, as if knives were running through it, and more so of late than in the beginning ginning of her diſorder. At firſt ſhe felt ſome pain in making water, but not lately: her mother thought that ſhe was unable to retain her urine long after the inclination to void it came on. In general ſhe did not ſuffer ſo much as to prevent her uſing a good deal of exerciſe, except on horſeback, which gave her conſiderable uneaſineſs. She otherwiſe enjoyed a pretty good ſtate of health.

Having received this account from her friends, I examined the patient, and found a ſubſtance appearing juſt without and filling up the os externum: it was of a red colour, and of a ſoftiſh, ſpongy texture, with an irregular, jagged ſurface; was ſore when touched; and a bloody ſerum oozed from it. It was ſomewhat contracted at its baſe, which was evidently attached anteriorly to the groove a little below the clitoris, its poſterior part lying looſe on the poſterior part of the os externum and entrance of the vagina; it could not be, therefore, a prolapſus uteri. While the labia were held aſunder by an aſſiſtant, I raiſed the fungus towards the pubis over a bent probe, which gave me an opportunity of examining its poſterior part, and of knowing that the meatus urinarius was not behind it; and by deſiring the patient to make make water, which paſſed through the center of the fungus, and by introducing a probe through the opening into the urethra, I was ſatisfied that the excreſcence ſprang from the whole circumference of the meatus urinarius, or extremity of the urethra itſelf; from which it appeared to be extended about half an inch. The cuticle, or membrane of the vagina, did not ſeem to be continued farther than where the urethra naturally terminates. The mother told me that the ſurface of the tumour at firſt was not jagged, but ſmooth.

I gave the friends my opinion, that the tumour was not formed by the uterus; that it was an excreſcence; and that a cure could not be expected but from extirpation. This they were deſirous of having performed; but being apprehenſive, on account of the cloſe connexion of the fungus with the urethra, that ſome troubleſome ſymptoms might ariſe in conſequence of the operation, I deſired them firſt to take farther advice. After ſome deliberation, they choſe to have the caſe repreſented to the late Mr. Donne, of Bath. He concurred with me in the neceſſity of the operation; he thought that much trouble would not occur afterwards; ſaid that a tent of lint dipped in oil might be introduced introduced into the urethra; but did not confider it as very neceffary, obferving that the urine would of itfelf keep the paffage open.

I ordered a gentle purge to be taken on the day preceding the operation, which was performed on the 23d, and in which I had the affiftance of the late Mr. Cooper, Surgeon at Wotten-Underedge, with whom I was at that time in partnerfhip.

The patient having made water, and being conveniently placed and fecured, a ligature was paffed through the body of the fungus, by means of a large crooked needle, and a loop formed, by which I was enabled to draw the fungus a little forwards and keep it fteady; and the border of the fungus being occafionally held afide with a hook, I was enabled the more eafily and expeditioufly to remove the fungus, which was carefully done with a very fmall knife, the incifion being made as near as poffible to that line, which feemed to diftinguifh the fungus from the urethra, by the termination of the cuticle or membrane of the vagina. The bleeding, after continuing pretty freely for a few minutes, ftopped of itfelf. A bit of lint fqueezed out of olive oil, and retained by a pledgit of white cerate, comprefs, and and bandage, was all the dreffing. The patient was put to bed, and directed to be kept on a cooling liquid diet.

On examining the fungus after the operation, it appeared about the fize of the nipple of an adult; its anterior part being expanded, formed, as it were, a little cup, with its border indented like a cock's comb, having a hole in its bottom, which was the orifice of the urethra, which ran through the body of the fungus: the internal membrane of the urethra was continued to the edge of the indented border, which was of a deeper red colour and fofter texture than the other parts of it. Before the operation the meatus urinarius was covered by the fides of the border folding over each other.

The patient made water foon after fhe was put to bed, and with but little fmarting; and again twice fhe voided a little urine fome time afterwards. In the afternoon, four or five hours after the operation, fhe began to be very uneafy from ftrangury; and two or three hours later I found her in great pain and uneafinefs from a conftant inclination to make water, without being able to void a drop. Her pulfe was quick; fhe looked rather hot; there was a confiderable tumour above the os pubis; nothing appeared appeared amiſs about the wound, which was of a darkiſh red colour, and the adjacent parts did not ſeem to be much ſwelled, although the meatus urinarius could not be diſtinctly ſeen. For three hours flannels wrung out of an emollient fomentation were applied to the pudenda and hypogaſtrium; ſhe took of a mixture of nitre and ſpermaceti, and drank freely of barley water with gum arabic. At ten in the evening, the pain increaſing, and the tumour from the diſtention of the bladder riſing very faſt, ſo as to reach almoſt to the navel, and being very hard and ſore to the touch, ſhe was put into a ſemicupium, in which ſhe ſat ten minutes; during which time, and for a few minutes afterwards, ſhe was free from pain, but could not void a drop of urine. I then introduced a ſmall catheter, without pain or difficulty *, and drew off a pint and a half of urine, highly tinged with blood. The diſtention of the bladder was entirely removed; ſhe received immediate eaſe, and preſently fell aſleep. In about three hours ſhe awaked with an inclination and

* Although I could not diſtinctly diſcern the orifice of the urethra, the groove below the clitoris and the wound proved a ſure guide for the catheter.

inability inability to make water. I immediately drew off the ſame quantity of urine as before, which was leſs tinged with blood. The catheter met with a ſlight obſtruction at the meatus urinarius. From this time to the 27th ſhe made water without the leaſt difficulty, and, excepting a ſlight ſoreneſs of the part, had no complaint. The dreſſing was renewed as often as neceſſary, to prevent any inconvenience from its being wet with urine.

On the 24th, the day after the operation, the urine was of a natural colour; the blood, with which it was before tinged, flowed moſt probably from the wound into the bladder. The orifice of the urethra was now obſervable.

On the 26th the wound was of a very little more florid colour than the adjacent parts. Obſerving a little riſing on one ſide of the meatus urinarius, not larger however than the fourth part of a grain of wheat, which ſeemed to be an unevenneſs left by the knife in the operation, but being fearful it might increaſe in bulk, I thought it prudent to touch it with the lunar cauſtic, and apply dry lint. She was permitted to ſit up, and to eat a little ſolid food.

On the 27th ſhe felt more ſmarting in making water; the part was more ſore, and ſeemed a little a little inflamed, which I attributed to the cauſtic, and thought it would ſoon go off; but very early the next morning a meſſenger was diſpatched for me in great haſte, on account of her having a return of the ſuppreſſion. She had paſſed very little urine from the time I was with her, about noon of the preceding day, and for ſome hours had been in great pain. I found, by the tumour in the hypogaſtrium, that the bladder was diſtended as much as in the former ſuppreſſion.

As ſhe had ſo readily obtained relief before from the catheter, after other means had proved ineffectual, I attempted, without heſitation, to introduce it now; but the obſtruction at the meatus was ſo great, that, with the degree of force which I thought I might venture to uſe, I could not overcome it. The attempt gave very great pain, and was followed by a conſiderable bleeding from the parts, which were much ſwollen: the bleeding, however, ſoon ceaſed. This diſappointment gave me and the friends of my patient much uneaſineſs.

As ſhe had been two days without a ſtool, I threw up an emollient clyſter, which ſoon produced a copious alvine dejection; but the ſuppreſſion of urine continued. She was ſoon after placed in the ſemicupium, in which ſhe continued fifteen minutes, and was eaſy.

While theſe things were doing, I revolved the caſe much in my mind: in particular, I conſidered how eaſily the catheter paſſed when I firſt uſed it immediately after her coming out of the ſemicupium, and that it did not paſs quite ſo eaſily ſome hours afterwards. Encouraged by this reflection, I hoped that I might again ſucceed after the ſemicupium; and therefore reſolved to make the attempt, and to leave the catheter in the bladder, if it ſhould ſucceed. For this purpoſe I cut off the catheter, ſo as to make a canula of a convenient length, and by ſome little contrivances prepared it for its being ſecured in the urethra. The ſuppreſſion not being at all relieved by the ſemicupium, I introduced the canula, which met with ſome reſiſtance at the meatus, but occaſioned no hæmorrhage, and drew off a pint and a half or a quart of urine, with the ſame relief as before. A cork was put in the canula, which was ſecured from ſlipping by tapes faſtened to a bandage round the waiſt. I directed that ſhe ſhould be kept very quiet in bed, lying on her back; that the canula ſhould not be removed, unleſs it occaſioned pain; and that the cork

ſhould be taken out when ſhe had an inclination to make water.

As the friends of the patient imputed this return of the ſuppreſſion to her being indulged in ſitting up, and eating ſolid food, though ſparingly, ſhe was again confined to a liquid diet; but I then thought that it was chiefly owing to the irritation brought on by the uſe of the cauſtic on the 26th.

My patient ſlept from ſeven o'clock this morning (the 28th) till noon, when her mother drew off the urine by the canula, according to my direction; but ſoon afterwards complaining of uneaſineſs, and ſome coagulated blood coming from the part, the canula was removed. She was eaſy after the diſcharge of the blood till about ſeven in the evening, when I found her complaining of pain from inability to make water, and I imagined that recourſe muſt have been again had to the catheter. She ſuffered ſo much from the firſt attempt to introduce it in the morning, that ſhe had a dread of it: ſhe was, however, deſirous of uſing the ſemicupium; in which, after continuing about ten minutes, ſhe grew faint, and upon being taken out her limbs were convulſed; but almoſt immediately on being laid on the bed ſhe fell aſleep, and ſlept till three or four o'clock the next morning, when ſhe awoke much refreſhed, and made water.

In the forenoon of this day (the 29th) the parts appeared free from inflammation; the meatus urinarius was very diſtinct, and its border ſomewhat prominent, ſo that the extremity of the urethra had, in a conſiderable degree, acquired its natural appearance: the little prominence, which had been touched with the cauſtic, remained of the ſame ſize, but did not differ in colour from the adjacent parts. She had by this time made water twice or three times, and was very cheerful. We were, therefore, in hopes, that, by keeping her quiet in bed, confining her to a liquid diet, and occaſionally giving ſome laxative medicine, for ſome days, and by applying nothing capable of exciting pain, we might prevent a relapſe. I was indeed of opinion that it might be proper to make uſe of the canula or bougie, after a few days, when the wound might be ſuppoſed to be nearly healed.

On the 30th, the ſtream of urine appearing to be ſomewhat twiſted, by the little riſing on the ſide of the meatus being turned a little over the orifice, I applied a ſmall bit of fine ſponge over over the lint, fo as to turn its point outwards from the meatus, which anfwered the purpofe.

On the 1ft of June there was only a little mucous difcharge on the dreffing; the part appeared to be healed, and naturally moift. During two or three hours this afternoon fhe had an inclination to make water, which fhe voided only by drops, attended with a fmarting that afterwards went off. The day following, however, when I examined the part, I could not perceive any thing amifs.

She had not yet fat up for the laft fix days; her belly had been kept fufficiently open, and fhe had eaten nothing more folid than boiled bread, or toaft with her tea, and this only for a day or two. As fhe made no complaint this day, (June 2) I permitted her to fit up, and to eat a little white meat.

On the 3d the fuppreffion of urine returned. As fhe had had no ftool for a day or two, an emollient clyfter was thrown up, which returning alone, another of pure oil was given, and this likewife came off without any fæces. She foon afterwards, however, made water, and was eafy. There being no forenefs, inflammation, or perceivable obftruction, the little rifing on the fide of the meatus being directed forwards, I ſuſpected that the veſſels of the part might remain ſomewhat relaxed in conſequence of the previous inflammation, and therefore, diſcontinuing the dreſſing, threw ſome cold water on the part with a ſyringe, and afterwards a very weak ſolution of white vitriol in water, which, producing no uneaſineſs, I ordered to be repeated twice or thrice a day.

On the 4th I was informed that ſhe had had a ſtool the preceding afternoon, and another that morning, when ſhe had likewiſe paſſed ſome urine; that ſoon afterwards, the ſuppreſſion returned, and ſhe appeared to ſuffer more pain than on the day before, but that it went off in about three hours.

Being now ſatisfied that, whether the cauſtic, coſtiveneſs, &c., had at all contributed to the ſuppreſſion or not, its returns muſt be owing to ſome other cauſe, and conſidering its returning firſt in three or four days after the uſe of the catheter, then in forty eight, and now in twenty-four, hours, without the uſe of the catheter, I ſuppoſed it to be moſt probably a ſtricture of the extremity of the urethra. I mentioned this to the friends of the patient the next day, (the 5th) and recommended the uſe of the bougie; to which they were averſe, and hoped there would be be no occaſion for it, as ſhe was now pretty well. While I was talking with them my patient began to find a return of the ſuppreſſion, and drinking freely of barley water, in hopes of removing it, the bladder filled apace, and in about two hours the pain became very great, and the pudenda began to ſwell. Recourſe was again had to the ſemicupium, in which ſhe ſat ten minutes, and was eaſy; but the pain returned as ſoon as ſhe came out. I would have introduced the catheter immediately, but from her dread of it was obliged to decline it. I threw up ſome oil and opium into the rectum, which was immediately rejected. The bladder was now diſtended to the navel, the external parts prodigiouſly ſwelled, and her pain ſo exceſſive, that her averſion to the catheter abated. Preſently afterwards, as I was about to order the ſemicupium, which I intended to uſe again previouſly to the introduction of the catheter, ſhe was ſenſible of a little urine eſcaping; in a little time the bladder emptied itſelf, and ſhe was eaſy. Flannels wrung out of warm water were applied to aſſiſt the ſubſiding of the ſwelling of the pudenda, which went quite off in about half an hour. I now urged the neceſſity

 of of uſing the bougie; to which, after ſome heſitation, the friends conſented. A ſmall one was introduced almoſt without her knowledge, and ſecured from ſlipping; and directions were given for its farther uſe.

She kept the bougie in the urethra two hours the firſt day; and on the next two days, when larger ones were uſed, three or four hours. It was afterwards introduced in the evening after ſhe went to bed, and removed in the morning before ſhe aroſe, and in this manner continued about a week. It was then uſed every other night, and laſtly twice a week to the end of the month, when it was omitted. During the uſe of the bougie ſhe, at firſt, had pretty frequent calls to make water, and afterwards was obliged to make water almoſt immediately upon the inclination coming on, which ſymptom went quite off in about a fortnight after the bougie was laid aſide. From the day ſhe began the uſe of the bougie ſhe had not the leaſt return of the ſuppreſſion of urine. On the 12th ſhe rode out on horſeback with a pleaſure ſhe had till then never known, as, before the removal of the fungus, the moſt gentle pace of the horſe gave her pain. Being ſomewhat reduced by the pain and confinement ſhe had ſuffered, ſhe then took a decoction of bark. She ſoon recovered her ſtrength; and it was afterwards obſerved that ſhe grew faſter than ſhe had done before the operation. Five years afterwards ſhe had not perceived the leaſt complaint in the urethra; and I have reaſon to believe that ſhe has felt none to this day.

I had an opportunity of ſpeaking to the ſurgeon who was firſt conſulted in this caſe. He informed me that, when he ſaw it, it was a ſmall, ſmooth, round, protruding body, with a hole in its middle, and was reducible by the finger; and that, from its appearance, and from her mother's account of its being firſt obſerved after taking the purge, he ſuppoſed that what he ſaw was the os internum, the uterus being, as he imagined, forced down into the vagina: he acknowledged, however, that it would ſlip down by the ſide of the cork he had adviſed to be worn as a peſſary.

REMARKS.

Excreſcences from the orifice and from the canal of the female urethra, ſome of which laſt have acquired ſuch a ſize as to protrude through the meatus urinarius, are related by authors*; in moſt of the inſtances of which the patients ſuffered ſymptoms ſimilar to thoſe excited by a ſtone in the bladder. Morgagni† has alſo mentioned two inſtances where he found, on examining, for other purpoſes, the bodies after death, a projection of a portion of the internal membrane of the urethra, ſimilar to that of the vagina or rectum. Whether or not the ſubjects of them ſuffered any inconvenience from the projection during life he was unacquainted: it would ſeem, from the circumſtances he has mentioned, not to have been great. But I have not met with an inſtance upon record of the whole extremity of the urethra expanded into a fungous excreſcence, like that I have above related; nor has another ſuch ſince occurred in my practice. Whether it was formed like the two juſt alluded to is

* Morgagni, De Sedibus & Cauſis Morborum, ep. 42; art. 42.—Sharpe, Critical Inquiry, p. 162.—Warner, Caſes in Surgery, third edition, caſe 45.—Bromfeild, Chirurg. Obſ. Vol. II. p. 296.—Jenner, London Medical Journal, Vol VII. p. 160.

† Place cited; and ep. 50, art. 51; ep. 56, art. 21.

perhaps perhaps uncertain ; but the account given of its firſt appearance, and the examination after its removal, ſeem to render it probable.

From the hiſtory of the above caſe may be learned, firſt, How the ſurgeon who was firſt conſulted, and whoſe anatomical knowledge might be ſuppoſed ſufficient to guard him againſt ſuch a deception, was led into the error of miſtaking the orifice of the urethra for that of the uterus, which ſhould teach us caution in making our inquiries, and candour to make allowances for the errors of each other, whilſt "humanum eſt errare."

Secondly, The young ſurgeon, who has not met with better opportunities, may be enabled to form ſome judgment of the exertions of the bladder to overcome an obſtruction to the egreſs of the urine.

Thirdly, There cannot be a doubt that, had a tent been introduced into the urethra immediately after the operation, and continued, at proper intervals, for perhaps a fortnight or three weeks, the ſuppreſſion of urine, the only diſagreeable circumſtance that occurred, might have been prevented. I declined uſing it, partly from Mr. Donne's opinion, and partly from confidering the analogy of the operation, with refpect to the urethra, to the amputation of the penis; in fome cafes of which * none was ufed, and by which perhaps I was more influenced than by the inftance which fell under the notice of Le Dran †, in which, from the want of a canula, a fuppreffion of urine fucceeded the operation.

If it fhould be thought a matter of indiffe-rence whether a filver canula, or a common or hollow bougie, had been ufed, I would beg leave to obferve, that, in my opinion, a filver canula would have been lefs likely to have increafed the inflammation of the urethra confequent to the operation, and proved moft convenient for the firft two or three days; after which the common bougie might have been ufed. Perhaps, at this time, a canula made of elaftic gum would be preferred to the filver one, or might ferve the purpofes of both canula and bougie.

Fourthly, Does not the above cafe point out the propriety of the ufe of the warm bath,

* Warner's Cafes, cafe 39.—Gooch's Cafes and Remarks in Surgery, fecond edition, p. 271.

† Treatife of Operations, Englifh tranflation, p. 158.

immediately immediately previoufly to the introduction of the catheter, in all cafes where the urine is required to be drawn off, in which the canal of the urethra is ftraightened by inflammation or fpafm?

*Stroud Water,*

Auguft 5th, 1791.

